# The Role of Radiotherapy in Soft Tissue Sarcoma on Extremities With Lymph Nodes Metastasis: An IPTW Propensity Score Analysis of the SEER Database

**DOI:** 10.3389/fonc.2021.751180

**Published:** 2021-10-21

**Authors:** Xinzhu Qiu, Hongbo He, Hao Zeng, Xiaopeng Tong, Qing Liu

**Affiliations:** Department of Orthopaedics, Xiangya Hospital, Central South University, Changsha, China

**Keywords:** soft tissue neoplasms, extremities, lymph node, radiotherapy, prognosis

## Abstract

**Background:**

Soft tissue sarcomas on extremities with regional lymph nodes metastasis (STSE-RLNM) is a devastating situation. Optimizing therapeutic approaches is vital but hampered by a shortage of randomized trials. We used a population-level database to evaluate radiotherapy’s impact on sarcoma-specific survival (SSS) and overall survival (OS) for surgery for STSE-RLNM.

**Methods:**

We retrospectively screened data from the SEER database (2004–2015), and 265 patients with STSE-RLNM who received surgery, with (134) or without (131) radiotherapy, were enrolled in this study. A propensity-score-matched analysis with the inverse probability of treatment weighting (IPTW) Kaplan–Meier curve was created. The log-rank test and Cox regression analysis were performed to compare SSS and OS in patients with and without radiotherapy. Further analysis of radiotherapy time was conducted, and the Kaplan–Meier curve and the log-rank test were done. Landmark analysis was introduced to attenuate the immortal bias.

**Results:**

In the original unadjusted cohort, the radiotherapy + surgery group is associated with improved SSS [hazard ratio (HR), 0.66; 95% CI, 0.47–0.91; p = 0.011] and OS (HR, 0.64; 95% CI, 0.47–0.88; p = 0.006). This significant treatment effect was also noted in IPTW-adjusted Cox regression either on SSS (HR, 0.65; 95% CI, 0.45–0.93; p = 0.020) or on OS (HR, 0.64; 95% CI, 0.46–0.91; p = 0.013). The Kaplan–Meier curve and log-rank test showed that pre- and postoperative radiotherapy was not related to SSS (p = 0.980 or OS (p = 0.890).

**Conclusion:**

Radiotherapy and surgery has a significant benefit on the prognosis of patients with STSE-RLNM compared to surgery alone. These findings should be considered when making treatment decisions for them.

## Introduction

Soft tissue sarcomas (STS) are a heterogeneous group of mesenchymal origin tumors and constitute just 1% of all malignancies. STS can occur anywhere in the body, with the extremities being the most common primary, accounting for 60% of all the STS (1, 2). Regional lymph node metastasis (RLNM) of soft tissue sarcomas on extremities (STSE) is a rarely observed clinical process. The real incidence of this kind of disease is difficult to determine accurately. There are few outcome studies of patients with sarcoma with lymph node metastases, and most of these indicate inferior survival (3–5). Despite the poor prognosis, regional lymph node metastasis of soft tissue sarcomas in extremities (STSE-RLNM) has been rarely studied due to its low incidence (1).

Radiotherapy is an integral component of limb-sparing therapy for STSE, and the benefit of radiation is more apparent for high- than low-grade tumors. After appropriately delivered radiation and surgery, the local recurrence is expected to be <10% (6). However, whether adjuvant radiotherapy has benefits to the overall survival of STSE has been controversial. Previous extensive dataset analyses of the impact of local treatment on survival outcomes for STSE are not consistent. Some studies based on comprehensive population databases like Surveillance, Epidemiology, and End Results (SEER) (7, 8) and National Cancer Database (NCDB) (9–11) indicated a survival benefit using radiotherapy in the treatment of high-grade STSE. The other three SEER studies showed no survival benefit for radiotherapy (12–14). Few studies of radiotherapy in STSE-RLNM exist because of the low incidence of the disease (15). To our knowledge, the role of radiotherapy in STSE-RLNM has not been well explored in a study with a comparatively large sample size.

We retrieved related data from the SEER database to overview the radiotherapy’s effect in patients with ESTS-RLNM. Prognosis factors were further analyzed for this rare case.

## Materials and Methods

### Data Sources and Study Population

**Figure 1:**
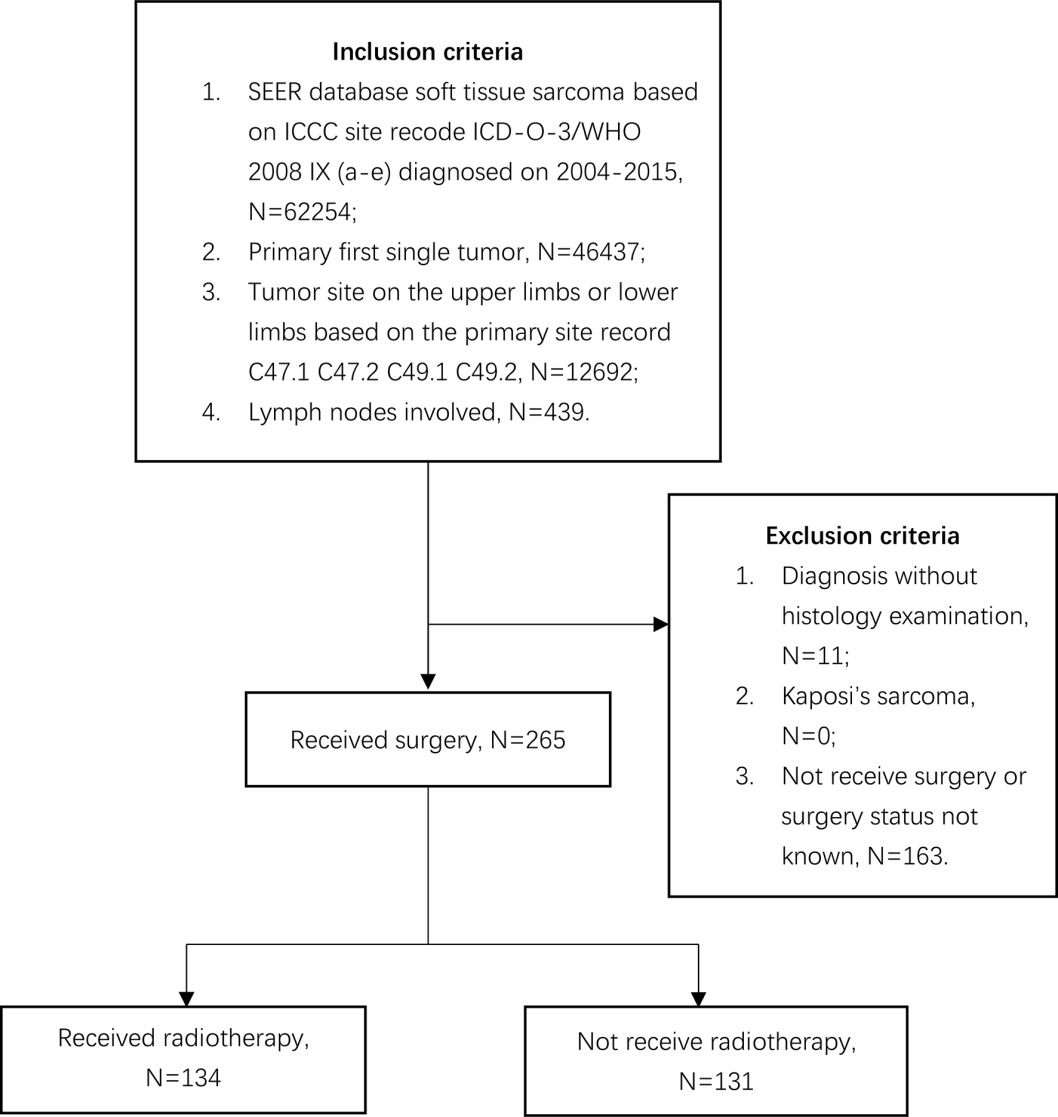
Flow diagram demonstrating the inclusion and exclusion criteria used to select soft tissue sarcoma of extremities with regional lymph nodes involved cases using the SEER 18 population-based cancer registry.

The SEER database, covering about 30% of the United States population, collects individual cancer data from 18 registries (16). The cancer data include no personal identifying information, and the National Cancer Institute permits data acquisition. We collected individual cancer data from this database *via* SEER*stat software version 8.3.8. We collected individual data on patients with STSE-RLNM from 2004 to 2015. Inclusion criteria were a diagnosis of soft tissue sarcoma on extremities based on primary site record C47.1, C47.2, C49.1, and C49.2 as the primary first single tumor with regional lymph nodes involved [American Joint Committee on Cancer (AJCC), 6th]. Data with Kaposi’s sarcoma, diagnosis without histology examination, and surgery not performed or unknown were excluded. The follow-up time was set as 12 years. The process of case selection can be illustrated in **Figure 1**.

### Covariates

We extracted data on sociodemographic, tumor-related, and treatment-related characteristics. Patients with STSE-RLNM were classified as those treated with and without radiotherapy. Tumor size was divided according to its influence on sarcoma-specific survival based on X-tile (17), and the most significant cutoff size is 150 mm. We divided tumor grades into two groups (I/II and III/IV) based on the degree of tumor differentiation. Based on codes in the SEER database, tumor stages were categorized into regional and distant. Primary endpoints were SSS and OS. SSS was considered as the time from diagnostic confirmation until death from STSE-RLNM. OS was considered as the period from the time from diagnosis to death due to any cause.

### Statistical Analysis

Differences in sociodemographic, tumor-related, and treatment-related characteristics between patients treated with surgery alone and surgery plus radiotherapy were assessed by Pearson’s chi-squared test and Fisher’s exact test. We introduced multiple imputation by chained equations (MICE) to deal with missing data on tumor size with relevant values (18, 19). A comparison of each variable was analyzed by univariable cox regression analysis. Variables reaching clinical significance (p < 0.05) were considered noted. Multivariable cox regression models were created by adjusting for variables selected from the univariate analysis.

Inverse probability of treatment weighting (IPTW) was used to balance the bias of confounding factors that may affect radiotherapy allocation (20). We calculated the standardized mean difference (SMD) to assess the balance of baseline characteristics after IPTW. Unadjusted and IPTW-adjusted Kaplan–Meier curves were drawn, and the treatments were analyzed with the log-rank test (21). Landmark analysis was introduced to attenuate immortal time bias (22). We then recreated IPTW-adjusted multivariable COX regression models and recalculated hazard ratios (HRs). Further analysis of the patients with radiotherapy was performed in a subgroup of radiotherapy time. We drew its Kaplan–Meier curves and compared radiotherapy time groups with the log-rank test.

All the statistical methods and graphs were done by R3.60. p <0.05 that was two-sided was defined as statistical significance.

## Results

### Characteristics Associated With the Use of Radiotherapy

**Figure 2:**
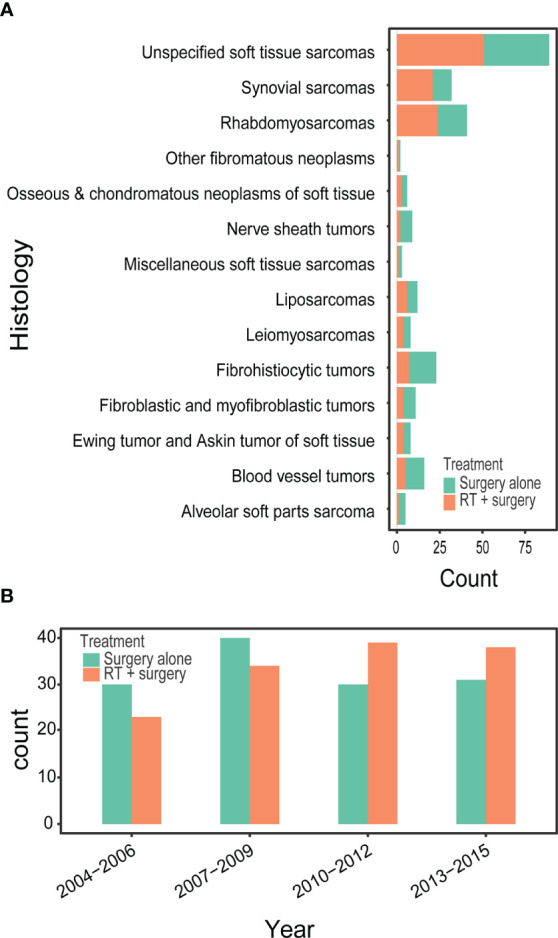
Bar diagram illustrating the treatment for patients with RSTS-RLNM in different histology **(A)** and over different diagnosis year **(B)** in an original unmatched cohort from the SEER database.

**Figure 3:**
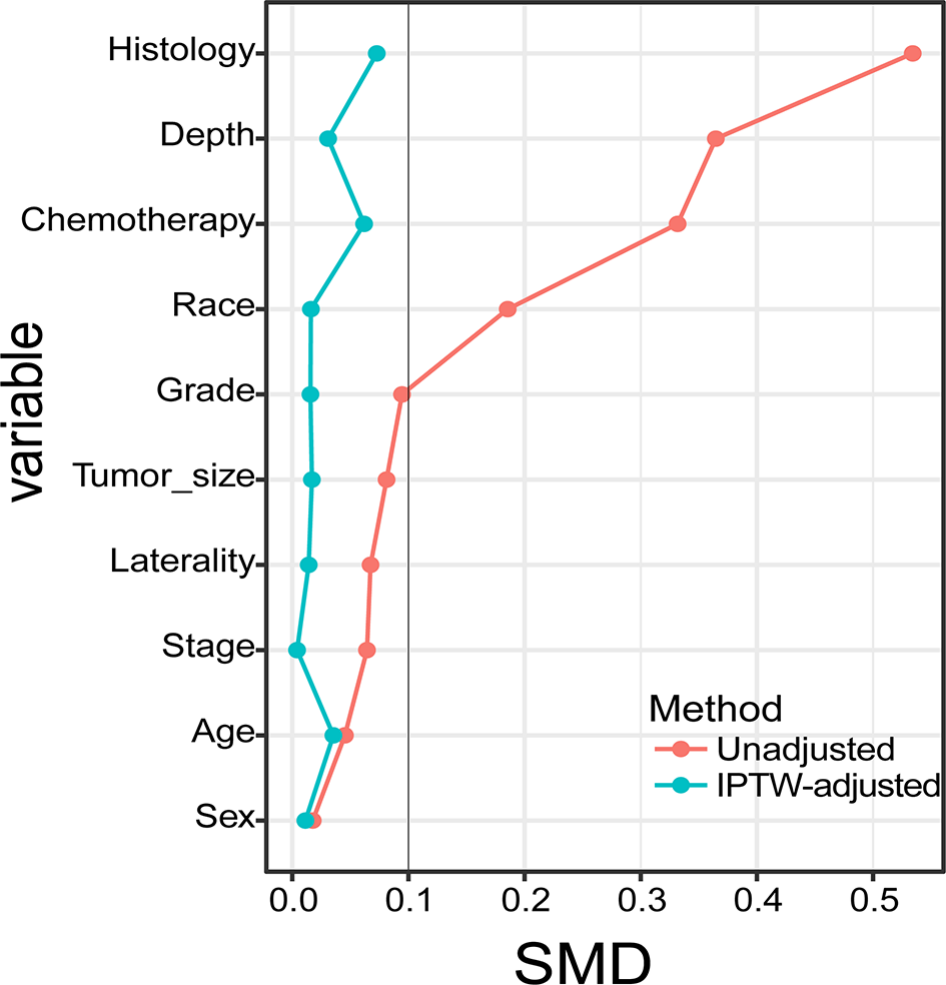
Standardized mean differences of different cohorts are presented in this graph.

Between 2004 and 2015, we identified 265 patients with STSE-RLNM who were histologically confirmed as having the primary malignancy with regional lymph nodes involved. A total of 131 (49.4%) received surgery alone, and 134 (50.6%) received radiotherapy and surgery. Baseline characteristics of the original population with STSE-RLNM are shown in **Table 1**. The number of patients receiving radiotherapy plus surgery was at a rising trend, and the proportion of different treatment groups also shows an escalating trend (**Figure 2B**). Patients received a nearly equal proportion of treatment in most kinds of histology (**Figure 2A**). Based on the propensity score, IPTW achieved an optimal balance between the two treatment groups. The SMD value all under 0.1 (**Figure 3**), indicating that confounding factors’ bias was attenuated.

**Table 1:** Sociodemographic and clinical characteristics of study patients.

	Unadjusted cohort			IPTW-adjusted cohort		
	Surgery alone	RT+ surgery	P	Surgery alone	RT+ surgery	P
N	131	134				
Age			0.830			0.792
<20	25 (19.1%)	28 (20.9%)		20.8%	22.3%	
≥20	106 (80.9%)	106 (79.1%)		79.2%	77.7%	
Race			0.172			0.904
Other	40 ()	30 (22.4%)		27.5%	26.8%	
White	91 (69.5%)	104 (77.6%)		72.5%	73.2%	
Sex			0.987			0.932
Female	50 (38.2%)	50 (37.3%)		34.9%	35.5%	
Male	81 (61.8%)	84 (62.7%)		65.1%	64.5%	
Grade			0.772			0.993
I/II	11 (8.4%)	11 (8.2%)		8.0%	8.2%	
III/IV	76 (58.0%)	72 (53.7%)		59.1%	58.4%	
Unknown	44 (33.6%)	51 (38.1%)		32.9%	33.3%	
Laterality			0.671			0.915
Left	66 (50.4%)	63 (47.0%)		50.2%	50.9%	
Right	65 (49.6%)	71 (53.0%)		49.8%	49.1%	
Histology			0.163			0.999
Alveolar soft parts sarcoma	4 (3.1%)	1 (0.7%)		1.3%	1.1%	
Blood vessel tumors	11 (8.4%)	5 (3.7%)		5.8%	4.9%	
Ewing and Askin sarcoma	4 (3.1%)	4 (3.0%)		2.9%	3.5%	
Fibroblastic & myofibroblastic tumors	7 (5.3%)	4 (3.0%)		4.1%	4.0%	
Fibrohistiocytic tumors	16 (12.2%)	7 (5.2%)		7.5%	7.4%	
Leiomyosarcomas	4 (3.1%)	4 (3.0%)		3.9%	3.6%	
Liposarcomas	6 (4.6%)	6 (4.5%)		5.4%	5.3%	
Miscellaneous soft tissue sarcomas	2 (1.5%)	1 (0.7%)		0.9%	1.1%	
Nerve sheath tumors	7 (5.3%)	2 (1.5%)		1.9%	2.2%	
Osseous and chondromatous sarcomas	3 (2.3%)	3 (2.2%)		3.1%	2.8%	
Other fibromatous neoplasms	1 (0.8%)	1 (0.7%)		0.9%	0.9%	
Rhabdomyosarcomas	17 (13.0%)	24 (17.9%)		16.2%	17.7%	
Synovial sarcomas	11 (8.4%)	21 (15.7%)		11.5%	11.2%	
Unspecified soft tissue sarcomas	38 (29.0%)	51 (38.1%)		34.6%	34.3%	
Stage			0.692			0.975
Regional	80 (61.1%)	86 (64.2%)		61.8%	62.0%	
Distant	51 (38.9%)	48 (35.8%)		38.2%	38.0%	
Tumor size			0.600			0.901
<150 mm	91 (69.5%)	98 (73.1%)		67.5%	68.3%	
≥150 mm	40 (30.5%)	36 (26.9%)		32.5%	31.7%	
Depth			0.014			0.972
Deep	77 (58.8%)	85 (63.4%)		66.5%	65.0%	
Superficial	15 (11.5%)	27 (20.1%)		12.6%	13.0%	
Unknown	39 (29.8%)	22 (16.4%)		20.9%	22.0%	
Chemotherapy			0.011			0.643
No	62 (47.3%)	42 (31.3%)		40.5%	37.5%	
Yes	69 (52.7%)	92 (68.7%)		59.5%	62.5%	

### Treatment effects of Radiotherapy Plus Surgery *vs.* Surgery Alone on Survival in Different Cohorts

The 5-year OS rates were 37.38% *vs.* 25.58%, respectively, in the group of patients treated with radiotherapy plus surgery and surgery alone. Similarly, the 5-year SSS rates showed 38.40% *vs.* 28.36% respectively, in the two groups. After adjusting in IPTW, the difference remained either in 5-year OS rate (40.88% *vs.* 25.82%) or in 5-year SSS rate (42.46% *vs.* 29.11%) (**Table 2**).

**Table 2:** Three-year, five-year, ten-year survival rates and median survival time in the unadjusted cohort and IPTW-adjusted cohort.

		3-year survival rate	5-year survival rate	10-year survival rate	Median survival time
Unadjusted SSS	Surgery alone	37.09%	28.36%	20.05%	20 months
	RT + surgery	51.24%	38.40%	34.47%	39 months
Unadjusted OS	Surgery alone	34.30%	25.38%	17.95%	18 months
	RT + surgery	49.88%	37.38%	31.58%	36 months
IPTW-adjusted SSS	Surgery alone	39.30%	29.11%	18.15%	21 months
	RT + surgery	54.51%	42.46%	36.73%	43 months
IPTW-adjusted OS	Surgery alone	35.97%	25.82%	16.10%	20 months
	RT + surgery	52.49%	40.88%	32.65%	41 months

SSS, sarcoma-specific survival; OS, overall survival.

**Figure 4:**
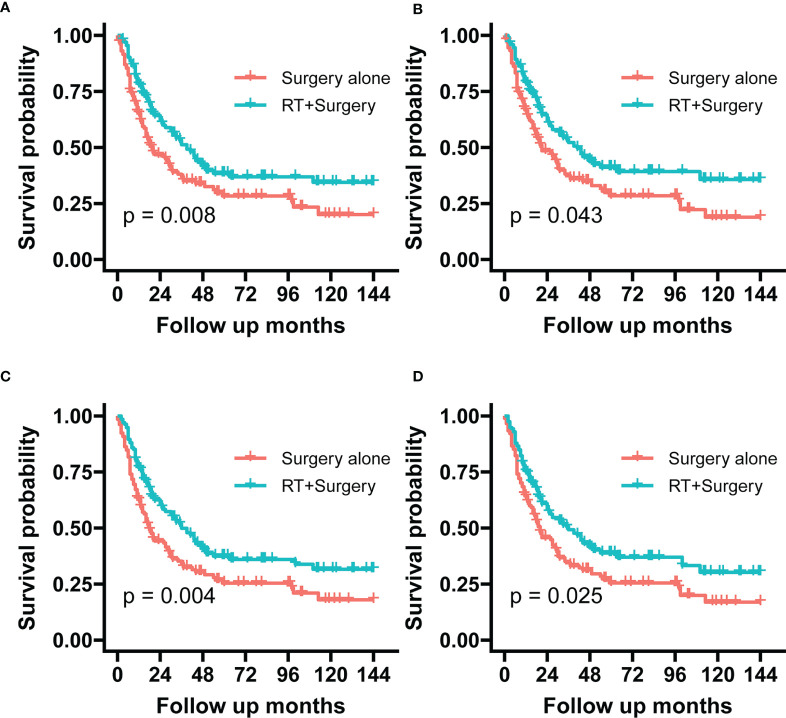
The graphs show Kaplan–Meier survival curves of sarcoma specific survival and overall survival in **(A, C)** unadjusted and **(B, D)** IPTW-adjusted cohorts of patients with STSE-RLNM.

**Figure 5:**
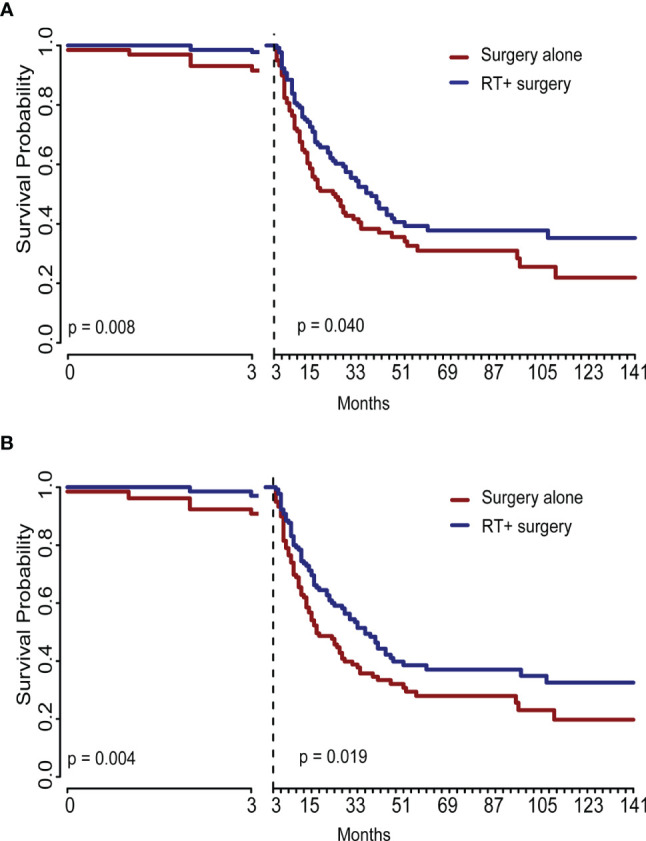
This graph showed the landmark analysis of the unadjusted original cohort. The difference between treatments remained significant at different periods either in sarcoma-specific survival **(A)** or in overall survival **(B)**. RT, radiotherapy.

The Radiotherapy plus surgery group showed a significantly superior survival benefit over surgery alone group either in the unadjusted cohort (OS, P = 0.004; SSS, (p = 0.008) or IPTW-adjusted cohort (OS, p = 0.025; SSS, p = 0.043) (**Figure 4**). A landmark analysis of the original cohort illustrated that immortal time bias was controlled and significance was still noted in radiotherapy’s effect on the two groups’ prognosis at different periods (**Figure 5**).

### Prognostic Characteristics for the Survival of STSE-RLNMs

In the univariable Cox regression model of the unadjusted cohort (**Table 3**), treatment with radiotherapy plus surgery reached statistical significance either in SSS [hazard ratio (HR), 0.66; 95% confidence interval (95% CI), 0.48–0.90; p = 0.009] or in OS (HR, 0.64; 95% CI, 0.48–0.8; p = 0.004). In multivariable Cox regression models, the significance remained in SSS (HR, 0.66; 95% CI, 0.47–0.91; p = 0.011) and in OS (HR, 0.64; 95% CI, 0.47–0.88; p = 0.006). After adjustment based on IPTW, the superior benefits of radiotherapy plus surgery are still significant either in SSS (univariable Cox regression: HR, 0.65; 95% CI, 0.46–0.91; p = 0.012; multivariable Cox regression: HR, 0.65; 95% CI, 0.45–0.93; p = 0.020) or in OS (univariable Cox regression: HR, 0.64; 95% CI, 0.46–0.89; p = 0.008; multivariable Cox regression: HR, 0.64; 95% CI, 0.46–0.91; p = 0.013). The characteristics selected in the univariable Cox regression model (p < 0.05) including age, histology, stage, grade, tumor size, and radiotherapy in unadjusted cohort and in IPTW-adjusted cohort (**Tables 3**–**6**).

**Table 3:** Univariable Cox regression models for sarcoma-specific survival and overall survival in patients with STSE-RLNM in the unadjusted cohort.

		SSS		OS	
		HR [95% CI]	P	HR [95% CI]	p
Age	<20	Reference			
	≥20	1.81 [1.18, 2.77]	0.007	1.78 [1.18, 2.69]	0.006
Race	Other	Reference			
	White	0.86 [0.61, 1.21]	0.382	0.77 [0.55, 1.06]	0.11
Sex	Female	Reference			
	Male	1.03 [0.75, 1.42]	0.847	1.06 [0.78, 1.45]	0.702
Grade	I/II	Reference			
	III/IV	2.53 [1.28, 5.01]	0.008	2.17 [1.17, 4.05]	0.015
	Unknown	1.69 [0.83, 3.43]	0.146	1.49 [0.78, 2.85]	0.226
Laterality	Left	Reference			
	Right	0.92 [0.68, 1.26]	0.606	0.91 [0.67, 1.23]	0.538
Histology	Liposarcoma	Reference			
	Alveolar soft parts sarcoma	3.59 [0.72, 17.82]	0.118	2.72 [0.61, 12.18]	0.191
	Blood vessel tumors	9.22 [2.62, 32.44]	0.001	7.45 [2.44, 22.71]	<0.001
	Ewing tumor and Askin tumor of soft tissue	1.66 [0.33, 8.21]	0.536	1.23 [0.28, 5.51]	0.785
	Fibroblastic and myofibroblastic tumors	3.80 [0.98, 14.73]	0.053	2.86 [0.83, 9.79]	0.095
	Fibrohistiocytic tumors	2.67 [0.76, 9.38]	0.125	2.17 [0.71, 6.59]	0.172
	Leiomyosarcomas	4.19 [1.05, 16.75]	0.043	3.16 [0.89, 11.21]	0.075
	Miscellaneous soft tissue sarcomas	2.04 [0.21, 19.63]	0.538	1.53 [0.17, 13.76]	0.702
	Nerve sheath tumors	5.47 [1.30, 23.06]	0.021	4.95 [1.39, 17.65]	0.014
	Osseous and chondromatous neoplasms of soft tissue	2.96 [0.66, 13.22]	0.156	2.22 [0.56, 8.89]	0.259
	Other fibromatous neoplasms	6.27 [1.05, 37.66]	0.045	4.74 [0.87, 25.99]	0.073
	Rhabdomyosarcomas	2.50 [0.75, 8.37]	0.136	2.06 [0.71, 5.95]	0.181
	Synovial sarcomas	3.73 [1.11, 12.53]	0.033	2.95 [1.01, 8.59]	0.047
	Unspecified soft tissue sarcomas	4.01 [1.25, 12.82]	0.019	3.24 [1.18, 8.93]	0.023
Stage	Regional	Reference			
	Distant	2.10 [1.54, 2.87]	<0.001	2.05 [1.51, 2.77]	<0.001
Tumor_size	<150 mm	Reference			
	≥150 mm	1.88 [1.36, 2.59]	<0.001	1.83 [1.33, 2.50]	<0.001
Depth	Deep	Reference			
	Superficial	1.11 [0.74, 1.69]	0.61	1.02 [0.67, 1.53]	0.937
	Unknown	0.91 [0.62, 1.34]	0.624	0.88 [0.60, 1.27]	0.488
Radiotherapy	No	Reference			
	Yes	0.66 [0.48, 0.90]	0.009	0.64 [0.48, 0.87]	0.004
Chemotherapy	No	Reference			
	Yes	0.87 [0.63, 1.20]	0.396	0.84 [0.62, 1.14]	0.252

**Table 4:** Multivariable Cox regression models for sarcoma-specific survival and overall survival in patients with STSE-RLNM in the unadjusted cohort.

		SSS		OS	
		HR [95% CI]	P	HR [95% CI]	P
Age	<20	Reference			
	≥20	2.81 [1.67, 4.73]	<0.001	2.74 [1.66, 4.53]	<0.001
Histology	Liposarcoma	Reference			
	Alveolar soft parts sarcoma	4.49 [0.84, 23.94]	0.078	3.37 [0.70, 16.17]	0.129
	Blood vessel tumors	10.32 [2.80, 38.05]	<0.001	8.43 [2.63, 27.03]	<0.001
	Ewing tumor and Askin tumor of soft tissue	2.71 [0.52, 14.16]	0.239	2.04 [0.43, 9.63]	0.369
	Fibroblastic and myofibroblastic tumors	5.16 [1.29, 20.63]	0.02	3.95 [1.12, 13.95]	0.033
	Fibrohistiocytic tumors	2.33 [0.63, 8.53]	0.203	1.93 [0.60, 6.15]	0.267
	Leiomyosarcomas	2.95 [0.70, 12.35]	0.139	2.29 [0.62, 8.50]	0.217
	Miscellaneous soft tissue sarcomas	2.88 [0.29, 28.72]	0.368	2.18 [0.24, 20.22]	0.493
	Nerve sheath tumors	5.93 [1.39, 25.41]	0.016	5.43 [1.50, 19.71]	0.01
	Osseous and chondromatous neoplasms of soft tissue	3.34 [0.74, 15.12]	0.117	2.45 [0.61, 9.89]	0.209
	Other fibromatous neoplasms	9.10 [1.40, 59.16]	0.021	7.02 [1.19, 41.57]	0.032
	Rhabdomyosarcomas	5.42 [1.51, 19.46]	0.009	4.51 [1.45, 14.06]	0.009
	Synovial sarcomas	5.09 [1.45, 17.91]	0.011	4.08 [1.34, 12.47]	0.014
	Unspecified soft tissue sarcomas	4.44 [1.35, 14.58]	0.014	3.68 [1.30, 10.45]	0.014
Stage	Regional	Reference			
	Distant	2.29 [1.61, 3.25]	<0.001	2.24 [1.60, 3.16]	<0.001
Grade	I/II	Reference			
	III/IV	2.35 [1.12, 4.92]	0.024	2.02 [1.03, 3.98]	0.041
	Unknown	1.80 [0.83, 3.90]	0.139	1.60 [0.78, 3.26]	0.198
Tumor_size	<150 mm	Reference			
	≥150 mm	1.40 [0.97, 2.03]	0.076	1.35 [0.94, 1.93]	0.101
Radiotherapy	No	Reference			
	Yes	0.66 [0.47, 0.91]	0.011	0.64 [0.47, 0.88]	0.006

**Table 5:** Univariable Cox regression models for sarcoma-specific survival and overall survival in patients with STSE-RLNM in the IPTW-adjusted cohort.

		SSS		OS	
		HR [95% CI]	P	HR [95% CI]	p
Age	<20	Reference			
	≥20	1.88 [1.22, 2.90]	0.005	1.83 [1.21, 2.78]	0.004
Race	Other	Reference			
	White	0.82 [0.58, 1.17]	0.279	0.71 [0.52, 0.98]	0.039
Sex	Female	Reference			
	Male	1.02 [0.72, 1.44]	0.924	1.07 [0.77, 1.50]	0.683
Grade	I/II	Reference			
	III/IV	2.52 [1.29, 4.93]	0.007	2.02 [1.14, 3.57]	0.016
	Unknown	1.88 [0.93, 3.80]	0.081	1.54 [0.84, 2.81]	0.163
Laterality	Left	Reference			
	Right	0.96 [0.68, 1.35]	0.81	0.94 [0.68, 1.31]	0.73
Histology	Liposarcoma	Reference			
	Alveolar soft parts sarcoma	1.98 [0.23, 16.84]	0.532	1.42 [0.19, 10.60]	0.731
	Blood vessel tumors	11.40 [3.26, 39.85]	<0.001	9.00 [3.26, 24.85]	<0.001
	Ewing tumor and Askin tumor of soft tissue	1.49 [0.25, 8.83]	0.658	1.04 [0.20, 5.35]	0.965
	Fibroblastic and myofibroblastic tumors	3.98 [1.15, 13.69]	0.029	2.82 [1.02, 7.78]	0.046
	Fibrohistiocytic tumors	2.22 [0.64, 7.75]	0.209	1.82 [0.65, 5.10]	0.252
	Leiomyosarcomas	4.32 [1.04, 17.97]	0.044	3.07 [0.89, 10.60]	0.076
	Miscellaneous soft tissue sarcomas	1.04 [0.07, 15.10]	0.975	0.74 [0.06, 9.68]	0.819
	Nerve sheath tumors	2.89 [0.56, 14.99]	0.207	2.49 [0.60, 10.41]	0.211
	Osseous and chondromatous neoplasms of soft tissue	3.43 [0.91, 12.98]	0.069	2.42 [0.78, 7.46]	0.124
	Other fibromatous neoplasms	6.89 [1.63, 29.10]	0.009	4.90 [1.40, 17.18]	0.013
	Rhabdomyosarcomas	2.33 [0.73, 7.48]	0.154	1.86 [0.75, 4.66]	0.183
	Synovial sarcomas	4.23 [1.34, 13.36]	0.014	3.24 [1.32, 7.93]	0.01
	Unspecified soft tissue sarcomas	4.68 [1.56, 14.07]	0.006	3.58 [1.55, 8.31]	0.003
Stage	Regional	Reference			
	Distant	2.10 [1.49, 2.95]	<0.001	2.04 [1.47, 2.83]	<0.001
Tumor_size	<150 mm	Reference			
	≥150 mm	2.06 [1.48, 2.86]	<0.001	1.92 [1.40, 2.64]	<0.001
Depth	Deep	Reference			
	Superficial	1.12 [0.69, 1.80]	0.652	1.01 [0.63, 1.62]	0.978
	Unknown	0.77 [0.49, 1.19]	0.237	0.75 [0.50, 1.13]	0.169
Radiotherapy	No	Reference			
	Yes	0.65 [0.46, 0.91]	0.012	0.64 [0.46, 0.89]	0.008
Chemotherapy	No	Reference			
	Yes	0.84 [0.59, 1.20]	0.335	0.81 [0.57, 1.14]	0.227

**Table 6:** Multivariable Cox regression models for sarcoma-specific survival and overall survival in patients with STSE-RLNM in the IPTW-adjusted cohort.

		SSS		OS	
		HR [95% CI]	P	HR [95% CI]	P
Age	<20	Reference			
	≥20	2.64 [1.48, 4.70]	0.001	2.55 [1.48, 4.40]	0.001
Grade	I/II	Reference			
	III/IV	2.64 [1.26, 5.57]	0.01	2.02 [1.01, 4.06]	0.048
	Unknown	2.32 [1.06, 5.08]	0.035	1.81 [0.88, 3.73]	0.109
Histology	Liposarcomas	Reference			
	Alveolar soft parts sarcoma	3.06 [0.48, 19.68]	0.239	2.17 [0.34, 13.63]	0.409
	Blood vessel tumors	12.05 [4.58, 31.67]	<0.001	9.81 [3.76, 25.60]	<0.001
	Ewing tumor and Askin tumor of soft tissue	2.43 [0.45, 13.21]	0.305	1.70 [0.31, 9.44]	0.545
	Fibroblastic and myofibroblastic tumors	4.97 [1.82, 13.56]	0.002	3.62 [1.36, 9.60]	0.01
	Fibrohistiocytic tumors	1.89 [0.69, 5.19]	0.218	1.61 [0.59, 4.41]	0.355
	Leiomyosarcomas	2.99 [0.89, 10.00]	0.076	2.18 [0.66, 7.27]	0.204
	Miscellaneous soft tissue sarcomas	1.62 [0.12, 21.89]	0.718	1.14 [0.09, 14.96]	0.919
	Nerve sheath tumors	2.71 [0.51, 14.47]	0.244	2.45 [0.50, 11.90]	0.267
	Osseous and chondromatous neoplasms of soft tissue	3.95 [1.45, 10.73]	0.007	2.58 [0.87, 7.66]	0.087
	Other fibromatous neoplasms	8.31 [2.70, 25.60]	<0.001	6.16 [2.09, 18.11]	0.001
	Rhabdomyosarcomas	4.67 [1.67, 13.06]	0.003	3.80 [1.40, 10.33]	0.009
	Synovial sarcomas	5.25 [2.13, 12.90]	<0.001	4.03 [1.66, 9.79]	0.002
	Unspecified soft tissue sarcomas	4.77 [2.07, 11.01]	<0.001	3.81 [1.68, 8.66]	0.001
Stage	Regional	Reference			
	Distant	2.32 [1.52, 3.56]	<0.001	2.30 [1.55, 3.41]	<0.001
Tumor_size	<150 mm	Reference			
	≥150 mm	1.45 [0.95, 2.20]	0.083	1.34 [0.90, 1.99]	0.152
Radiotherapy	No	Reference			
	Yes	0.65 [0.45, 0.93]	0.02	0.64 [0.46, 0.91]	0.013

In the multivariable Cox regression analysis adjusted for related characteristics for OS rate in the original unadjusted cohort (**Table 3**), age ≥20 (HR, 2.74; 95% CI, 1.66–4.53; p < 0.001), stage of distant metastasis (HR, 2.24; 95% CI, 1.60–3.16; p < 0.001), grade of III/IV (HR, 2.02; 95% CI, 1.03–3.98; p = 0.041), the histology of blood vessel tumors (HR, 8.43; 95% CI, 2.63–27.03; p < 0.001), fibroblastic and myofibroblastic tumors (HR, 3.95; 95% CI, 1.12–13.95; p = 0.033), nerve sheath tumors (HR, 5.43; 95% CI, 1.50–19.71; p = 0.010), rhabdomyosarcomas (HR, 4.51; 95% CI, 1.45–14.06; p = 0.009), and synovial sarcomas (HR, 4.08; 95% CI, 1.34–12.47; p = 0.014) were all reported as independent prognostic factors for OS of STSE-RLNM (**Table 4**). Independent prognostic factors reported in the multivariable Cox regression models in IPTW-adjusted cohort remained the same (**Table 6**). Likewise, prognostic factors for OS rate remained statistically significant for SSS rate in patients with STSE-RLNM (**Tables 4**, **6**).

### Further Investigation in the Subgroup of Radiotherapy Time

**Figure 6:**
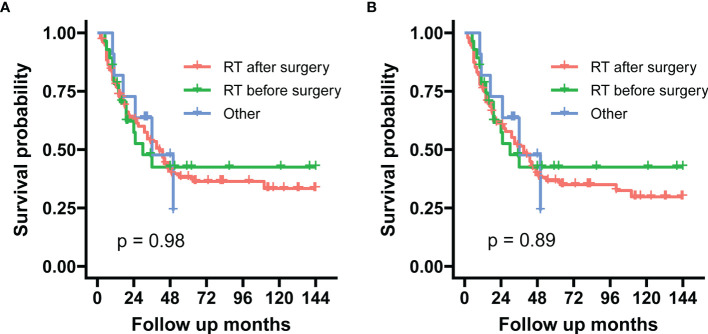
These graphs show Kaplan–Meier survival curves of sarcoma-specific survival **(A)** and overall survival **(B)** in the unadjusted cohort between preoperative and postoperative radiotherapy.

Within the subgroup of radiotherapy time, preoperative radiotherapy *vs.* postoperative radiotherapy showed no difference in the log-rank test in both SSS (p = 0.980) and OS (p = 0.890) (**Figure 6**).

## Discussion

In recent decades, because of the extremely low incidence and a limited number of studies, the optimal treatment strategy of STSE-RLNM is controversial. Surgery is regarded as the mainstay for STS, which has improved the prognosis, as reported in many studies (3, 23). Radiation therapy combined with surgery has been reported to achieve better prognosis than surgery alone in intermediate and high-grade STS (11, 24). Nevertheless, little was known about the effect of radiation therapy of STSE-RLNM, and a few studies reported no benefit of radiation therapy in OS of STSE-RLNM (25, 26). The negative results of treatment with radiation therapy may be explained by the relatively small sample size and related studies bias. To address the limitations mentioned above, we conducted IPTW-adjusted analysis of a sample of 265 patients with STSE-RLNM treated with surgery. We found that the survival rate (both OS and CSS) improved significantly in the group receiving radiation therapy plus surgery. Several independent prognostic factors for STSE-RLNM were also identified through multivariable Cox regression analysis. There are some meaningful findings in the current study.

This study represents large cohorts of STSE-RLNM with 265 patients included based in the SEER database from 2004 to 2015. Because of the low incidence of STSE-RLNM, most published studies concerning STSE-RLNM are based on small sample size (15, 25, 26). Most tumors are high grade (grade III/IV), consistent with previous studies with a relatively large sample of STSE-RLNM (25, 27). Our study’s median overall survival months (18 months in surgery alone cohort and 36 months in RT + surgery cohort) is longer than previous reviews (12.7 months) probably because our patients all suffered from surgery. During different times, the ratio of radiation therapy has shown a relatively escalating trend, which may indicate doctors’ increasing tendency to use radiation therapy as an adjuvant method for surgery. Based on the IPTW calculated according to all the covariables that mislead treatment allocation, allocation bias was attenuated mainly (20). The significant treatment effect of adjuvant radiotherapy in the original cohort was also noted in the adjusted groups based on IPTW. There have been some studies concerning the treatment of STSE in which adjuvant radiotherapy has a protective effect on the prognosis in high-risk patients (13, 28–32). In the field of STSE-RLNM, studies are rare. A SEER analysis containing 1,597 patients found that the survival of STSE-RLNM has nothing to do with adjuvant radiotherapy and chemotherapy (25). However, the number of patients who got lymph nodes involved was only 28, and the results were not adjusted by propensity score match. Other studies that got negative radiotherapy results also have the defects of low sample size and did not adjust the correlative factors (15, 26). We further studied the effect of radiation sequence on the survival of the patients with STSE-RLNM. No difference was found in the current study through log-rank analyzing of the survival curve of radiation therapy prior to surgery and after surgery. Our results were consistent with several retrospective studies on STS (33–36). Despite its similar survival rates, the time of radiotherapy is related to different complications. As previous studies indicate, pre- and postoperative radiotherapy were associated with higher wound complications rates (35, 36) and higher long-term toxicity of radiotherapy (including joint stiffness, muscle fibrosis, etc.) rates (37, 38), respectively.

Although radiotherapy is recommended for treating STSE-RLNM in our research, it is necessary to consider some late effects of radiotherapy when specifically applied to the clinic. Iqbal et al. (39) recruited 34 patients with soft tissue sarcoma who received radiotherapy and found that 12 patients (35%) developed acute lymphedema, and 22 patients (65%) developed chronic lymphedema, suggesting that reserving a reasonable amount of lymphatic retention should be considered when performing radiotherapy on patients with STS. However, patients with STSE-RLNM also need to clean the lymph as much as possible to avoid further tumor progression. To grasp the balance between the two requires clinicians to conduct individualized analysis and use appropriate radiotherapy methods. Proton radiotherapy is the current cutting-edge method in the field of radiotherapy. Compared with traditional photon radiotherapy, protons can better concentrate the radiation energy on the tumor target area to be treated to protect normal organs and tissues. Therefore, the tumor can be adequately irradiated under relatively small doses of radiotherapy, and local tumor control can be improved. At the same time, radiation complications of normal organs and tissues can be greatly reduced. Thomas et al. (40) reported that the long-term toxicity, short-term toxicity, and the incidence of secondary tumors of proton radiotherapy are lower than photon therapy. Proton radiotherapy may light the path of treating STSE-RLNM patients.

Tumor immunotherapy is a treatment that enables the body to raise a tumor-specific immune response through active or passive ways and exert its function of inhibiting and killing tumors. At present, the clinical indications of immunotherapy are more and more extensive, and it has become the leading treatment for metastatic diseases (41). Despite that the field cancer immunotherapy was founded by clinicians caring for sarcoma patients, the response of immunotherapy for sarcoma varies because of its various histological origin and immune microenvironment (40, 42). A phase II trial of pembrolizumab in patients with selected STSs and bone sarcomas was carried out by Tawbi et al. (43). They found that the response rate is 18% in STS, 40% in undifferentiated pleomorphic sarcoma, 20% in liposarcoma, and 10% in synovial sarcoma. Adjuvant radiotherapy combined with immunotherapy may increase response rates for some kinds of sarcoma. Finkelstein et al. (44) recruited 17 patients with high-grade STS. Then, these patients were treated with radiotherapy combined with intratumoral injection of DSs. Nine of 17 (52.9%) patients developed immune responses, and 12 of 17 (70.6%) patients were progression-free after 1 year. There are also some ongoing immunotherapy studies in STSs. A phase I trial tests the role of combining ipilimumab, nivolumab, and radiation in resectable soft tissue sarcoma (NCT03463408). Another phase I/II study involving patients with high-risk soft-tissue sarcoma evaluated the efficacy of neoadjuvant durvalumab and tremelimumab plus radiation (NC T03116529). We look forward to more research on soft tissue sarcoma immunotherapy combined with radiotherapy, which may bring more benefits to STSE-RLNM patients.

In the current study, we also found that the older age, grade III/IV in pathology, and distant metastasis were significantly related to poor prognosis of STSE-RLNM. This study showed a higher survival rate either overall or sarcoma-specific in the young population than their older counterparts, which is according to previous studies (45, 46). This difference is likely due to the pre-existing comorbidities and reduced physical and psychological reserves of elders (47). A high grade in pathology and distant metastasis were related to poor prognosis, and STSE-RLNM is no exception. The histology was introduced in the current studies, and similar to other studies on STS (whether lymph nodes involved or not) (48–53), liposarcoma was associated with better prognosis compared to other histology, and on the contrary, blood vessel tumors, fibroblastic and myofibroblastic tumors, nerve sheath tumors, rhabdomyosarcomas, and synovial sarcomas showed a relatively poor prognosis. Hiroyuki Tsuchie et al. (54) studied soft tissue sarcoma with distant metastasis and found malignant peripheral nerve sheath tumor (MPNST) had a poor prognosis than myxoid liposarcoma, which also confirmed our result. Compared with surgery alone, chemotherapy combined with surgery showed no significant benefit of survival. The results should be interpreted with caution, since we did not study chemotherapy’s single role in STSE-RLNM. As chemotherapy plays an essential tool in patients with unresectable tumors or distant metastases (55), that part of chemotherapy needs further research.

The study also has some limitations in both design and data. First, detailed information about radiotherapy, including radiation dose, and the specific radiation site was not recorded in the SEER database. Therefore, we could not conclude the effects of radiotherapy stratified by dose and the particular radiation site. Second, some unobserved cofounders, including specific surgical types, whether lymph nodes resected or not, distant metastatic sites, and tumor necrosis rates, may affect prognosis. Nevertheless, through systematic multiple analysis of nine tumor-related and treatment-related covariables, radiotherapy’s significant treatment effect was stable across all the cohorts. Third, we mainly concentrated on the OS and SSS in this study. Further study could include other aspects, such as quality of life, treatment-related complications treatment costs, and comprehensively assessing patient status. Finally, this study is retrospective because of the properties of the SEER database. However, it is impossible to conduct randomized controlled trials (RCTs) of STSE-RLNM because of the disease’s epidemiological and clinical characteristics.

## Conclusions

Our study first indicated that compared to surgery alone, surgery combined with radiotherapy could improve the SSS and OS of STSE-RLNM. In addition, adjuvant radiotherapy was an independent protective factor for the prognosis of STSE-RLNM. Besides, we found that older age, higher grade, distant metastasis, and histology including malignant blood vessel tumors, synovial sarcomas, rhabdomyosarcomas, fibroblastic, and myofibroblastic tumors were independent risk factors of STSE-RLNM. Our findings encourage the application of radiotherapy for patients with STSE-RLNM.

## Data Availability Statement

The datasets presented in this study can be found in online repositories. The names of the repository/repositories and accession number(s) can be found below: SEER database.

## Ethics Statement

The studies involving human participants were reviewed and approved by the Ethics Committee of Xiangya Hospital, Central South University. Written informed consent for participation was not required for this study in accordance with the national legislation and the institutional requirements.

## Author Contributions

XQ and QL designed the study. XQ, QL, HH, HZ, and XT analyzed the data. XQ, QL, and HH wrote the manuscript. XQ and QL reviewed the manuscript. All authors contributed to the article and approved the submitted version.

## Funding

This work was supported by the Hunan Youth Science and Technology Innovation Talent Project (2020RC3058) and the Research project of Hunan health and Family Planning Commission (C20180785).

## Conflict of Interest

The authors declare that the research was conducted in the absence of any commercial or financial relationships that could be construed as a potential conflict of interest.

## Publisher’s Note

All claims expressed in this article are solely those of the authors and do not necessarily represent those of their affiliated organizations, or those of the publisher, the editors and the reviewers. Any product that may be evaluated in this article, or claim that may be made by its manufacturer, is not guaranteed or endorsed by the publisher.

## Abbreviations

STSE-RLNM, soft tissue sarcomas on extremities with regional lymph nodes metastasis; SSS, sarcoma-specific survival; OS, overall survival; STS, soft tissue sarcomas; RLNM, regional lymph node metastasis; STSE, soft tissue sarcomas on extremities; SEER, Surveillance, Epidemiology, and End Results; NCDB, National Cancer Database; MICE, multiple imputation by chained equations; IPTW, inverse probability of treatment weighting; SMD, standardized mean difference; HR, hazard ratios; MPNST, malignant peripheral nerve sheath tumor; RT, radiotherapy.
